# Modulators of Protein Kinase C Affect SR-BI-Dependent HDL Lipid Uptake in Transfected HepG2 Cells

**DOI:** 10.1155/2011/687939

**Published:** 2011-01-05

**Authors:** Rachelle Brunet, Maxine How, Bernardo L. Trigatti

**Affiliations:** Atherothrombosis Research Group, Department of Biochemistry and Biomedical Sciences, Thrombosis and Atherosclerosis Research Institute, Hamilton Health Sciences, McMaster University, 1200 Main St. W. Hamilton, ON, Canada L8N 3Z5

## Abstract

SR-BI is a cell surface HDL receptor that mediates selective uptake of the lipid cargo of HDL, an important process in hepatocytes, driving reverse cholesterol transport from cells in the artery wall. To facilitate examination of factors that modulate SR-BI activity in hepatocytes, we have generated fluorescent protein-tagged versions of SR-BI that allow for facile monitoring of SR-BI protein levels and distribution in transfected cells. We show that deletion of the C-terminal cytosolic tail does not affect the distribution of SR-BI in HepG2 cells, nor is the C-terminal cytosolic tail required for SR-BI-mediated uptake of HDL lipids. We also demonstrate that the phorbol ester, PMA, increased, while protein kinase C inhibitors reduced SR-BI-mediated HDL lipid uptake in HepG2 cells. These data suggest that protein kinase C may modulate selective uptake of HDL lipids including cholesterol in hepatocytes, thereby influencing hepatic HDL cholesterol clearance and reverse cholesterol transport.

## 1. Introduction

High-density lipoproteins (HDL) mediate reverse cholesterol transport from cells in the artery wall to the liver where cholesterol and cholesteryl ester are taken up by the scavenger receptor class B type 1 via a process known as selective lipid uptake [1]. This is the uptake of the lipid components of the HDL particle without the net internalization and degradation of the particle itself [2]. Reverse cholesterol transport (RCT) driven by SR-BI in the liver, therefore, represents a key pathway for hepatic clearance of HDL cholesterol and prevention of the build-up of cholesterol in inflammatory cells in the artery wall, thereby protecting against atherosclerosis [3, 4]. Studies from gene-targeted mouse models have demonstrated that knocking out SR-BI expression results in impaired hepatic clearance of HDL cholesterol leading to increased amounts of cholesterol in the blood associated with abnormally large HDL particles, as well as reduced levels of cholesterol in bile [5–11]. On the other hand, overexpression of SR-BI in livers of mice results in increased clearance of HDL cholesterol and is accompanied by reduced levels of cholesterol associated with HDL in blood, and increased levels of cholesterol in bile [12–14]. 

Epidemiological studies have revealed that higher levels of HDL cholesterol in blood are associated with protection and that lower levels of HDL cholesterol in blood are associated with increased risk for atherosclerosis leading to heart disease [15]. In light of this, the finding that eliminating SR-BI expression increases while its overexpression reduces atherosclerosis in mice [6, 8, 16–19] may appear surprising. The results can be reconciled by considering the activity of RCT as the important atheroprotective factor rather than the absolute level of HDL cholesterol in the blood. Thus, knocking out SR-BI expression in mice results in impaired RCT due to a lack of expression of SR-BI in livers [20]. This leads to the appearance of unusually large, cholesterol laden HDL particles because cholesterol cannot be cleared from HDL by selective uptake. This accumulation of cholesterol in HDL, in turn, reduces the ability of HDL to remove excess cholesterol from the artery wall, thereby increasing the development of atherosclerosis. On the other hand, overexpressing SR-BI in livers of mice results in increased RCT activity, characterized by an increase in the clearance of HDL cholesterol from blood, and leads to reduced steady-state blood HDL cholesterol levels. The increased RCT activity also increases clearance of cholesterol by HDL from cells in the artery wall, resulting in reduced development of atherosclerosis. This has been supported by studies that demonstrate the effects of manipulating SR-BI expression on HDL cholesterol clearance, steady-state levels of HDL cholesterol in blood and cholesterol in bile, and on the development of atherosclerosis in mouse model systems [5–14, 16–20]. Understanding factors that regulate SR-BI activity will therefore shed light on pathways that may regulate RCT activity in vivo.

SR-BI is a 509 amino acid protein that is heavily glycosylated and embedded in the plasma membrane via two transmembrane domains, close to the N- and C-termini of the protein [4]. SR-BI contains two cytosolic regions, one of approximately 10 amino acids at its N-terminus and the other of ~40 amino acids at its C-terminus [4]. The terminal 3-4 amino acids of the C-terminal cytosolic domain represents a binding site for an adaptor protein called PDZK1 which plays an important role in protecting SR-BI protein from degradation in hepatocytes [21–23]. The precise sequences that direct SR-BI towards degradation in the absence of PDZK1 binding remain to be identified; however it is presumed that they reside in the C-terminal cytosolic tail of SR-BI. PDZK1 may thus represent one mechanism by which SR-BI protein levels can be adjusted to regulate RCT activity. Indeed PDZK1 has been shown to be phosphorylated by protein kinase A, which appeared to be necessary for its ability to stabilize SR-BI [24]. PDZK1's role in stabilizing SR-BI, however, is tissue specific since elimination of PDZK1 in mice results in the virtual absence of SR-BI protein in hepatocytes, only partially reduced levels in intestinal cells, and no observed effects on other tissues [25, 26]. 

We have previously reported that the protein kinase C (PKC) activators, phorbol esters, can increase, while specific PKC inhibitors can reduce SR-BI-dependent lipid uptake in transfected Chinese hamster ovary cells overexpressing SR-BI [27]. This was accompanied by opposite effects on the level of cell association of the HDL particles themselves indicating that PMA activates and PKC inhibitors reduce SR-BI-dependent selective lipid uptake activity. The role of PKC activity on SR-BI-mediated lipid uptake in hepatocytes, however, is not known. Here we demonstrate that SR-BI overexpression in HepG2 cells mediates increased cellular HDL lipid uptake. Using this system, we demonstrate that PMA enhances and PKC inhibitors suppress SR-BI-mediated HDL lipid uptake in hepatocytes. This sensitivity to PKC inhibitors is not affected by the removal of the cytosolic C-terminus of SR-BI, nor does it involve alterations in the level of SR-BI expression or distribution.

## 2. Materials and Methods

### 2.1. Cell Culture and Transfection

Expression plasmids for full-length human SR-BI and SR-BI-ΔC (lacking the C-terminal cytosolic tail) fused at their N-termini to either enhanced green fluorescent protein (eGFP) or momeric red fluorescent protein (mRFP) have been described previously [28]. For transfection, HepG2 cells were seeded at a density of 5 × 10^5^ cells per well in 6-well dishes and cultured in Dulbecco's modified eagle's medium containing 5% fetal bovine serum, 2 mM L-glutamine and penicillin (50 U/ml) and streptomycin (50 *μ*g/ml). On the following day, 3 *μ*L of Fugene 6 transfection reagent and 97 *μ*L of serum-free medium were mixed and incubated at RT for 5 min prior to the addition of 1 *μ*g of plasmid DNA and further gentle mixing. After incubation for a further 20 min, the mixture was added to one well containing 2 mL of fresh growth medium. The dishes were rocked back and forth and side to side to ensure even distribution of the transfection solution. The cells were cultured for 48 hrs prior to use for experiments. 

To select for stably expressing cells, the transfected HepG2 cells were cultured in growth medium containing 500 *μ*g/mL of G418 beginning 24 hrs after transfection. After 4 days, cells selected with G418 were further sorted individually into wells of a 96-well plate in order to isolate clonal cell lines. Sorted cells were cultured in growth medium containing 500 *μ*g/mL of G418. The medium was replaced with fresh medium every 2 days. Once the cells could be seen in each well, eGFP expression was analyzed by fluorescence wide-field microscopy using an Axiovert 200M inverted fluorescence microscope (Carl Zeiss Canada Inc). Isolates expressing eGFP were expanded, and expression eGFP and SR-BI was characterized by immunoblotting.

### 2.2. Fluorescence Microscopy

Cells cultured on poly-D-lysine coated, glass-bottomed dishes were fixed with freshly prepared 2.5% paraformaldehyde in phosphate-buffered saline (PBS). Cells were incubated for 4 min in 2 mL of 300 nM 4′-6-diamidino-2-phenylindole (DAPI) in PBS to stain nuclei. Stained cells were washed 3 times with PBS, and fluorescence microscopy was performed using an Axiovert 200M inverted fluorescence microscope (Carl Zeiss Canada Inc). Fluorescence images were captured with Zeiss filter sets Fs01 (excitation: band pass 365/12 and emission: long pass 397; DAPI imaging), Fs13 (excitation: band pass 470/20 and emission: band pass 505–530; eGFP imaging), and/or Fs15 (excitation: band pass 546/12 and emission: long pass 590; mRFP imaging).

### 2.3. SDS-PAGE and Immunoblotting

Cell lysates were prepared as previously described [27], and protein concentrations were determined using the BCA Protein Assay Kit (Pierce). Protein electrophoresis under denaturing conditions and immunoblotting were performed as previously described [28].

### 2.4. DiI-HDL Lipid Uptake Assay

HDL was prepared from human plasma by KBr ultracentrifugation and labelled with 1,1′-dioctadecyl-3,3,3′,3′-tetramethylindocarbocyanine perchlorate (DiI) as previously described [1, 29, 30]. On day 0, 5 × 10^5^ HepG2 cells were seeded per well of 6-well dishes. On day 2, the cells were washed once with 1 mL of incomplete DMEM. For experiments requiring the treatment of cells with various inhibitors or activators, the cells were incubated in incomplete DMEM containing 0.5% BSA and the inhibitor or activator at the appropriate concentration for 10 min at 37°C. Following these incubations, DiI-HDL was added to each well to a final concentration of 5 *μ*g/mL. Duplicate wells received 5 *μ*g/mL DiI-HDL and a 40-fold excess (200 *μ*g/mL) of unlabeled HDL to allow for the measurement of nonspecific cell association of DiI fluorescence. For experiments without treatments with inhibitors or activators, the DiI-HDL and unlabeled HDL were added immediately after the wash with incomplete DMEM at the final concentrations stated above. The plates were incubated at 37°C for 2 hrs. The cells were washed twice with cPBS (PBS containing 0.01% CaCl_2_ and 0.01% MgCl_2_) released from the dishes by trypsinization (0.05% trypsin, 0.53 mM EDTA in PBS) for 2 min, washed in ice-cold complete media, and resuspended in ice-cold PBS containing 0.1% BSA, 0.5 mM EDTA, and 0.1% glucose. The samples were analyzed for DiI and eGFP fluorescence using a Beckman Coulter Epics XL flow cytometer. Specific uptake of DiI from HDL (defined as the level of uptake that could be competed away by a 40-fold excess of unlabeled HDL) was determined as the difference between the mean DiI fluorescence of the cells incubated with DiI-HDL alone and the mean DiI fluorescence when cells were incubated with both DiI-HDL and a 40-fold excess of unlabeled HDL.

## 3. Results

**Figure 1:**
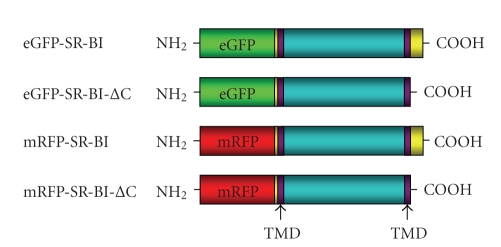
Diagrams of the eGFP or mRPF-tagged SR-BI and SR-BI-ΔC transgenes. The constructs consist of either SR-BI or SR-BI-ΔC tagged with either eGFP or mRFP on their amino termini. The locations of putative transmembrane domains (TMDs) are also shown. Cytosolic regions are shown in yellow and extracellular regions are shown in blue.

**Figure 2:**
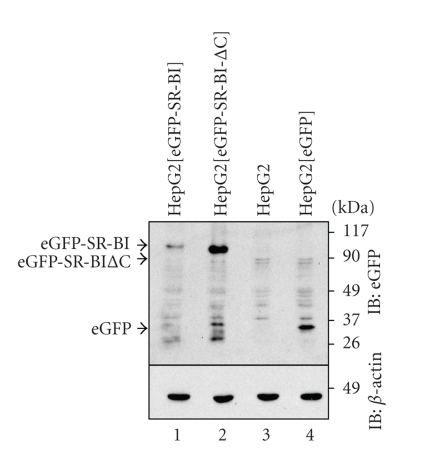
Expression of eGFP-SR-BI or eGFP-SR-BI-ΔC in HepG2 cell lines. Cell lysates prepared from HepG2[eGFP-SR-BI] (lane 1), HepG2[eGFP-SR-BI-ΔC] (lane 2), HepG2 (untransfected) (lane 3), and HepG2[eGFP] cells (lane 4) were subjected to SDS-PAGE and immunoblotting for eGFP (upper panel) or *β*-actin (lower panel).

We have generated versions of human SR-BI encoding the full-length polypeptide or a deletion mutant lacking the 40 amino acid C-terminal cytosolic tail (hereafter referred to as SR-BI-ΔC) and tagged on the N-terminal cytosolic region with either eGFP- or mRFP [28] (Figure 1). Tagging with fluorescent proteins allows the facile monitoring of the levels and distributions of the proteins expressed in transfected cells, and it allows us to distinguish the full-length and mutant forms of the protein in doubly transfected cells. We have previously demonstrated that tagging of SR-BI at the N-terminus with fluorescent proteins does not alter its distribution in cells or its lipid uptake activity [28]. To examine the involvement of the C-terminal cytoplasmic tail of SR-BI in its distribution and activity in hepatocytes, we transfected plasmids encoding the tagged wild-type and mutant versions of SR-BI into HepG2 cells and selected for stably expressing cells by a combination of drug resistance and fluorescence-activated cell sorting (not shown). As a control, we also generated HepG2 cells stably expressing eGFP alone.  Figure 2 shows the levels of eGFP-tagged full-length SR-BI or SR-BI-ΔC, or of eGFP alone in transfected and stably expressing HepG2[eGFP-SR-BI], HepG2[eGFP-SR-BI-ΔC], HepG2[eGFP] cells, or untransfected HepG2 cells, as analyzed by SDS-PAGE and detected by immunoblotting using an antibody against eGFP. No GFP immunoreactive band was detected in untransfected HepG2 cells as expected. Cells transfected and stably expressing eGFP alone contained a band migrating between the 26 and 37 kDa molecular weight markers, consistent with the expected 30 kDa size of eGFP. Lysates from HepG2[eGFP-SR-BI] cells contained a band migrating between the 90 kDa and 117 kDa molecular weight markers consistent with the expected size of 115 kDa (30 kDa eGFP + 85 kDa SR-BI). Lysates from HepG2[eGFP-SR-BI-ΔC] cells contained an eGFP immunoreactive band that migrated slightly faster than that in HepG2[eGFP-SR-BI] lysates, consistent with the expected size of eGFP fused to SR-BI lacking the C-terminal cytoplasmic tail. *β*-actin was used as a control to demonstrate equal loading across all lanes. Interestingly, deletion of the C-terminal cytosolic tail of SR-BI appeared to correspond to higher SR-BI protein levels (Figure 2, compare lanes 1 and 2). Results of flow cytometric analysis of HepG2[eGFP-SR-BI] and HepG2[eGFP-SR-BI-ΔC] cells (see Figure 5(b)) also suggested that truncation of the cytosolic C-terminus corresponded to higher SR-BI protein levels. We interpret this to be due to the removal of sequences in the C-terminal cytosolic tail of SR-BI that are responsible for its degradation in hepatocytes. It is known that the binding of PDZK1 to the cytosolic C-terminus of SR-BI is required to stabilize SR-BI in hepatocytes, since deletion of the PDZK1 binding site (the last 3 amino acids) from the cytosolic C-terminal tail of SR-BI or knockout of PDZK1 results in the degradation of SR-BI [22, 23, 25], presumably due to other sequences present in the C-terminal cytosolic tail itself. 

**Figure 3:**
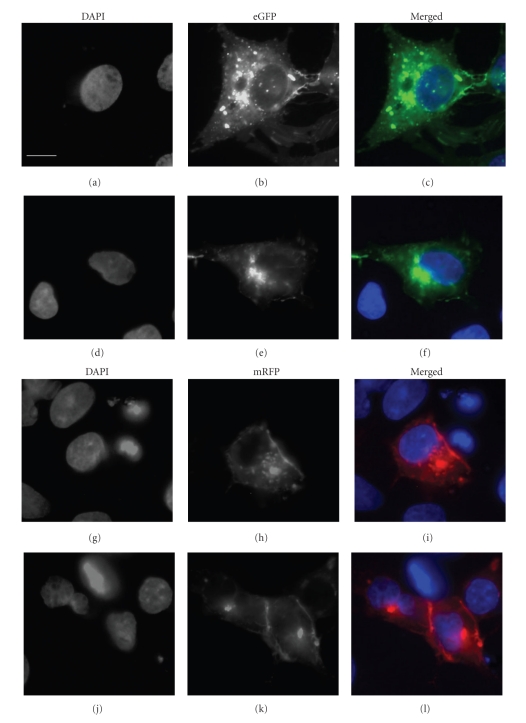
Localization of fluorescent protein-tagged SR-BI containing or lacking the C-terminal tail in transfected HepG2 cells. HepG2 cells transfected with eGFP-SR-BI ((a)–(c)), eGFP-SR-BI-ΔC ((d)–(f)), mRFP-SR-BI ((g)–(i)), or mRFP-SR-BI-ΔC ((j)–(l)) were fixed, stained with DAPI and imaged by wide field fluorescence microscopy in the blue ((a), (d), (g), and (j)), green ((b) and (e)), or red ((h) and (k)) channels. Merged images of the blue and green ((c) and (f)) or the blue and red ((i) and (l)) channels are also shown. All images are set to the same scale. Scale bar in (a) = 10 *μ*m.

To examine the effects of deletion of the C-terminal cytosolic tail of SR-BI on its distribution in HepG2 cells, we examined HepG2 cells transfected with expression plasmids encoding either eGFP-tagged or mRFP-tagged versions of full-length SR-BI or SR-BI-ΔC (Figure 3). Both eGFP-tagged or mRFP-tagged full-length SR-BI and SR-BI-ΔC exhibited similar subcellular distributions in HepG2 cells. This consisted of punctate staining over the body of cells with intense staining at regions of the cell periphery, particularly in areas of cell-cell contact. This corresponds to the distribution of SR-BI described previously in a variety of transfected cells or cells expressing endogenous SR-BI [27, 28, 31]. Similar results were seen when we examined stably expressing HepG2[eGFP-hSR-BI] and HepG2[eGFP-SR-BI-ΔC] cells (not shown). We also performed immunofluorescence microscopy on permeabilized HepG2[mRFP-SR-BI] cells using an antibody that binds to the C-terminus of SR-BI, which demonstrated colocalization of the immunofluorescence and the mRFP signals (data not shown). This confirmed that the fluorescent protein signals represented the distribution of the intact tagged SR-BI.

**Figure 4:**
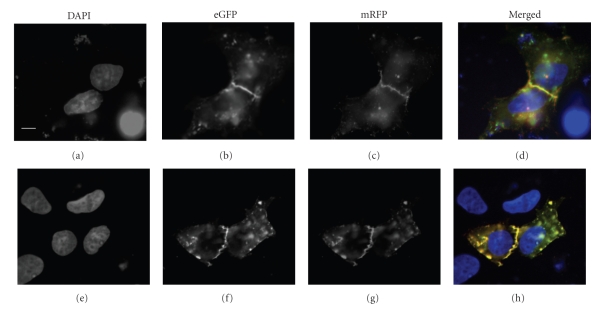
Colocalization of fluorescent protein-tagged SR-BI and SR-BI-ΔC in transfected HepG2 cells. HepG2 cells transfected with both eGFP-SR-BI and mRFP-SR-BI-ΔC ((a)–(d)) or with both mRPF-SR-BI and eGFP-SR-BI-ΔC ((e)–(h)) expression plasmids were fixed, stained with DAPI, and imaged by wide-field fluorescence microscopy in the blue ((a) and (e)), green ((b) and (f)), and red ((c) and (g)) channels for DAPI, eGFP, and mRFP, respectively. Merged images are shown in panels (d) and (h). All images are set to the same scale. Scale bar in (a) = 10 *μ*m.

To confirm that the deletion of the C-terminal cytosolic tail of SR-BI did not alter its distribution in HepG2 cells, we transfected HepG2 cells with both eGFP-SR-BI and mRFP-SR-BIΔC expression plasmids (Figures 4(a)–4(d)). This allowed us to examine the distribution of full-length SR-BI and SR-BI-ΔC in the same cells, by examining the distribution of the green and red fluorescent protein tags. Figures 4(b) and 4(c) demonstrate the similarity in the distribution of eGFP-SR-BI and mRFP-SR-BI-ΔC in the same cells. The merged image shown in Figure 4(d) demonstrates substantial colocalization of the green and red fluorescent protein signals, particularly at the cell periphery in the region of cell-cell contact. To confirm that the distributions of full length SR-BI or SR-BI-ΔC were not influenced by the fluorescent protein tag, we reversed the florescent protein tags by examining HepG2 cells cotransfected with plasmids encoding eGFP-SR-BI-ΔC and mRFP-SR-BI and observed similar results (Figures 4(e)–4(h)).

**Figure 5:**
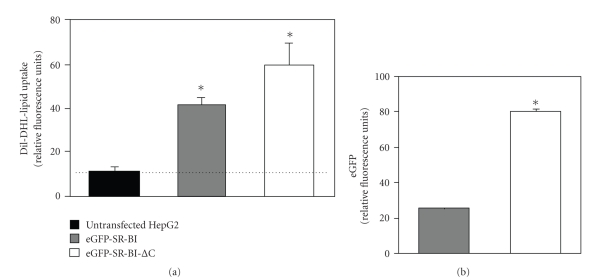
SR-BI-ΔC retains DiI-HDL lipid uptake activity. HepG2 cells and stably transfected HepG2[eGFP-SR-BI] and HepG2[eGFP-SR-BI-ΔC] cells were incubated with DiI-HDL and analyzed by flow cytometry for DiI fluorescence (a) or eGFP fluorescence (b). The specific uptake of DiI lipid is shown in panel (a). The geometric mean eGFP fluorescence for HepG2[eGFP-SR-BI] and HepG2[eGFP-SR-BI-ΔC] cells is shown in panel (b). The experiment was performed in triplicate, and error bars represent standard deviation. *P < .05 by the Student's *t*-test.

We tested the effects of deletion of the C-terminal cytosolic tail of SR-BI on its ability to mediate the uptake of lipids from HDL in HepG2 cells. Untransfected HepG2 cells or stably expressing HepG2[eGFP-SR-BI] cells or HepG2[eGFP-SR-BI-ΔC] cells were incubated with 100 *μ*g/ml HDL containing the fluorescent exchangeable lipid DiI for 2 hours at 37°C. Cells were then examined by flow cytometry for DiI fluorescence and for eGFP fluorescence (as a measure of the abundance of tagged SR-BI protein). Previous research from a number of labs has demonstrated that, despite the differences in the structures of DiI and cholesterol, DiI uptake from HDL is an effective surrogate for the uptake of HDL lipids including cholesterol [1]. Untransfected HepG2 cells exhibited very low DiI-HDL lipid uptake activity that was close to background (Figure 5(a)). In contrast, HepG2 cells stably expressing either eGFP-SR-BI or eGFP-SR-BI-ΔC exhibited substantially increased levels of DiI uptake from HDL, demonstrating that overexpression of either full-length SR-BI or SR-BI-ΔC confers increased HDL lipid uptake (Figure 5(a)). We noted significantly higher levels of DiI uptake in HepG2[eGFP-SR-BI-ΔC] cells than in HepG2[eGFP-SR-BI] cells (Figure 5(a)). This may be explained by the greater abundance of SR-BI in HepG2[eGFP-SR-BI-ΔC] cells than in HepG2[eGFP-SR-BI] cells as revealed by flow cytometric analysis of eGFP levels (Figure 5(b)) consistent with the results of immunoblotting described earlier (Figure 2).

**Figure 6:**
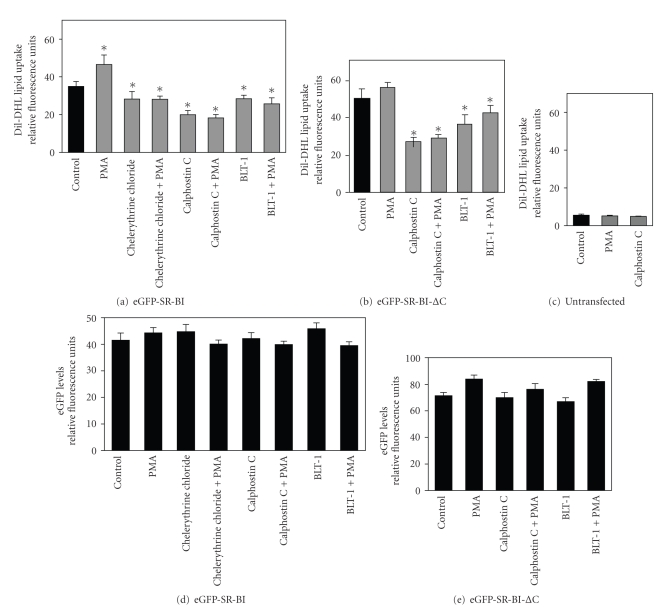
The protein kinase C inhibitor calphostin C reduces SR-BI-mediated DiI-HDL lipid uptake in HepG2 cells independently of SR-BI's C-terminal tail. DiI-HDL lipid uptake (panels (a)-(c)) and eGFP fluorescence (panels (d) and (e)) were measured by flow cytometry. HepG2[eGFP-SR-BI] cells untreated or treated with 0.5 *μ*M PMA, 7.5 *μ*M chelerythrine chloride, 1 *μ*M calphostin C, 300 nM BLT-1, individually or in combination as indicated panels (a) and (d). HepG2[eGFP-SR-BI-ΔC] cells untreated or treated with PMA, calphostin C, or BLT-1 either individually or in combination as indicated panels (b) and (e). Panel (c): untransfected HepG2 cells treated without or with PMA or calphostin C individually as above. The data represent average ± standard deviations of triplicate samples. *P < .05 compared to control by Student's *t*-test.

We have previously demonstrated that PMA, a phorbol ester activator of protein kinase C, increased and that a protein kinase C inhibitor reduced SR-BI-mediated selective HDL lipid uptake in transfected Chinese hamster ovary cells [27]. We therefore tested if activation or inhibition of protein kinase C affected SR-BI activity in hepatocytes overexpressing SR-BI. We treated HepG2[eGFP-SR-BI], HepG2[eGFP-SR-BI-ΔC], or untransfected HepG2 cells with PMA, to activate protein kinase C, or with the protein kinase C inhibitors calphostin C or chelerythrine chloride. As controls, HepG2 cells overexpressing SR-BI or SR-BI-ΔC were treated with the small molecule SR-BI inhibitor BLT-1, which is known to inhibit SR-BI dependent HDL lipid uptake [32]. After a 10 min pre-incubation, cells were incubated with DiI-HDL in the continued presence of PMA, and/or the PKC or SR-BI inhibitors for 2 hours at 37°C prior to flow cytometric analysis of the level of cell associated DiI fluorescence (Figure 6). Treatment of HepG2[eGFP-SR-BI] cells expressing full length SR-BI (Figure 6(a)) with PMA resulted in a statistically significant approximately 40% increase in DiI-HDL lipid uptake. In contrast, treatment of cells with the protein kinase C inhibitors, chelerythrin chloride or calphostin C, resulted in an approximately 25%–40% decreases in DiI-HDL lipid uptake when compared to control untreated cells. Treatment with the PKC inhibitors also completely prevented the PMA-mediated increase in DiI-HDL lipid uptake in HepG2[eGFP-SR-BI] cells. Similarly, treatment of cells with BLT-1, the small molecule inhibitor of SR-BI mediated selective HDL lipid uptake [33], resulted in a statistically significant ~20% reduction in HDL lipid uptake and also completely prevented the PMA mediated increase in HDL lipid uptake. Similar results were obtained when we tested HepG2[eGFP-SR-BI-ΔC] cells overexpressing the mutant form of SR-BI lacking the C-terminal cytosolic tail (Figure 6(b)), although, again, the level of HDL lipid uptake in these cells was greater than that measured in HepG2[eGFP-SR-BI] cells (compare black bars in panels (a) and (b)). In fact, PMA treatment did not further increase the high level of HDL lipid uptake in HepG2[eGFP-SR-BI-ΔC] cells, suggesting it may have reached a maximum. Nevertheless, treatment with the PKC inhibitor calphostin C or the SR-BI-specific inhibitor, BLT-1, reduced DiI-HDL lipid uptake in HepG2[eGFP-SR-BI-ΔC] cells (Figure 6(b)). The background level of DiI-HDL lipid uptake in untransfected HepG2 cells (Figure 6(c)) was substantially lower and was not substantially affected by either PMA or calphostin C treatment (Figure 6(c)). Therefore, the effects of PMA and calphostin C on HDL lipid uptake in HepG2[eGFP-SR-BI] and HepG2[eGFP-SR-BI-ΔC] cells are due to alterations in the activity of the overexpressed SR-BI. None of the treatments affected the levels of SR-BI protein as detected by eGFP fluorescence in HepG2 cells expressing either eGFP-hSR-BI or eGFP-SR-BI-ΔC (Figures 6(d) and 6(e)). These data indicate that PKC activity modulates HDL lipid uptake mediated by SR-BI in hepatocytes. 

**Figure 7:**
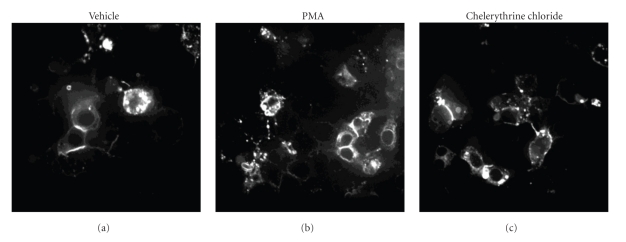
Modulators of PKC activity do not affect SR-BI distribution in HepG2 cells. HepG2[eGFP-SR-BI] cells were serum starved for 4 hrs and then refed medium containing 5% FBS and either vehicle (0.1% DMSO), 0.5 *μ*M PMA, or 7.5 *μ*M chelerythrine chloride and incubated for 1 hr. Cells were fixed with 2.5% paraformaldehyde and imaged by confocal microscopy.

SR-BI is located on the cell surface as well as intracellularly and continuously cycles between the two compartments in hepatocytes and a variety of other cell types [27, 28, 34–38]. Activation or inhibition of phosphatidyl inositol 3 kinase signaling has been reported to alter the distribution of SR-BI between the cell surface and internal compartments in hepatocytes and adipocytes [35, 39]. To determine if the effects of modulating PKC activity on SR-BI mediated HDL lipid uptake involved alterations in the distribution of SR-BI between the cell surface and internal compartments, we treated HepG2[eGFP-SR-BI] cells with either PMA, the PKC inhibitor chelerythrin chloride, or vehicle alone and imaged the cells by confocal fluorescence microscopy. Neither activation of PKC with PMA nor its inhibition with chelerythrine chloride resulted in detectable alterations in the subcellular distribution of eGFP-SR-BI in stably expressing HepG2 cells when compared to controls that were either untreated (not shown) or treated with DMSO vehicle alone (Figure 7). This suggests that neither the activation of PKC nor its inhibition alters the cell surface localization of SR-BI.

## 4. Discussion

We have utilized an approach in which SR-BI protein levels, distribution, and activity can easily be monitored in HepG2 cells in order to examine factors that may influence SR-BI function in hepatocytes and therefore the activity of RCT. This approach involves the expression of recombinant forms of SR-BI containing fluorescent protein tags on their amino termini. The N-terminus of SR-BI consists of a short 10 amino acid peptide that is predicted to protrude into the cytosol. Fusion of fluorescent proteins to the N-terminus of SR-BI does not appear to alter its distribution in cells (Figures 3 and 4) or its ability to mediate HDL binding or HDL lipid uptake (Figure 5) [28, 36, 40]. Therefore the use of SR-BI fused to fluorescent proteins represents a convenient method to simultaneously monitor effects on SR-BI protein levels, distribution, and activity. 

Using this system, we have demonstrated that the removal of the cytoplasmic C-terminus of SR-BI does not prevent it from mediating HDL lipid uptake, indicating that it is dispensable for this activity in hepatocytes. Similar findings have been reported by our group in Chinese hamster ovary derived cells [28] and by others in MCF-7 mammary epithelial cells [41]. We did, however, observe higher protein levels corresponding to the mutant version of SR-BI lacking the C-terminal cytoplasmic portion (eGFP-SR-BI-ΔC) compared to the full length SR-BI (eGFP-SR-BI) in transfected HepG2 cells. This is consistent with the C-terminal cytoplasmic region of SR-BI directing its degradation via a pathway that is prevented by PDZK1 binding [21–23, 25]. These findings suggest that the levels of full-length SR-BI protein in HepG2 cells overexpressing eGFP-SR-BI are limited by the availability of PDZK1 and that any amount of full-length SR-BI in excess of the available PDZK1 is degraded. On the other hand, eGFP-SR-BI-ΔC, which lacks the C-terminal cytoplasmic tail and therefore both the sequences that direct its degradation in the absence of PDZK1 and the PDZK1 binding site itself, is not limited by the availability of PDZK1, allowing higher levels of this protein to accumulate. Consequently, HepG2 cells overexpressing eGFP-SR-BI-ΔC exhibited higher levels of HDL lipid uptake than did HepG2 cells overexpressing eGFP-SR-BI (full length) (Figures 5 and 6) due to the higher SR-BI protein levels.

We have also demonstrated that treatment of HepG2 cells overexpressing eGFP-tagged SR-BI with PKC inhibitors decreased HDL lipid uptake. Conversely, treatment with PMA, a phorbol ester that activates PKC, increased HDL lipid uptake in cells expressing full-length SR-BI. Thus activation of PKC promotes while PKC inhibitors reduce SR-BI-mediated HDL lipid uptake in hepatocytes, consistent with our findings in transfected Chinese hamster ovary cells. Deletion of the cytoplasmic C-terminus of SR-BI did not prevent the ability of PKC inhibitors to suppress SR-BI-mediated HDL lipid uptake, suggesting that this effect likely does not reflect direct phosphorylation of SR-BI, at least in its cytosolic C-terminal tail. We also did not observe any alterations in the abundance of eGFP-tagged SR-BI in cells treated with either PMA or PKC inhibitors. This, along with the finding that the C-terminal cytoplasmic tail of SR-BI was not necessary, demonstrates that this is likely not through alterations in the phosphorylation and/or activity of PDZK1. Finally our results also suggest that activation or inhibition of PKC does not grossly affect the distribution of SR-BI in HepG2 cells. Thus, the effect of PKC activity is likely not through recruitment of SR-BI to the cell surface, as has been reported to be the consequence of phosphatidyl inositol 3 kinase signaling activity [35, 39]. 

Recently, Tall and coworkers reported that a novel compound, ITX5061, developed as a p38 mitogen-activated protein kinase (MAPK) inhibitor, inhibited SR-BI-dependent HDL lipid uptake in cultured cells overexpressing SR-BI and that treatment of mice with this compound resulted in reduced HDL cholesterol clearance from plasma and increased HDL size and cholesterol content [42]. This suggests that inhibitors of p38MAPK may also reduce hepatic SR-BI function and impair RCT. The P38 MAPK pathway has been shown to be activated by PKC in hepatocytes and hepatocellular carcinoma cells [43–47]. This raises the possibility that SR-BI-dependent HDL lipid uptake activity in hepatocytes is regulated by a pathway involving PKC-mediated activation of p38 MAPK signaling. However, the involvement of p38 MAPK signaling in PKC mediated activation of SR-BI-dependent HDL lipid uptake remains to be tested. Furthermore the downstream targets of regulation that mediated the increased SR-BI activity remain to be identified. Our studies suggest that regulation of SR-BI-dependent HDL lipid uptake by PKC does not involve the C-terminal cytoplasmic tail of SR-BI, the portion of the molecule that would be the most likely candidate for direct phosphorylation.

## Abbreviations

DAPI: 4′-6-diamidino-2-phenylindole
DiI: 1,1′-dioctadecyl-3,3,3',3'-tetramethylindocarbocyanine perchlorate
eGFP: Enhanced green fluorescent protein
HDL: High density lipoprotein
MAPK: Mitogen activated protein kinase
mRFP: Monomeric red fluorescent protein
PBS: Phosphate buffered saline
PDZ: Postsynaptic density protein (PSD-95)/ *Drosophila* disc large tumor suppressor (dlg)/ tight junction protein (ZO1)
PKC: Protein kinase C
PMA: Phorbol 12-myristate 13-acetate
RCT: Reverse cholesterol transport
SR-BI: Scavenger receptor, class B, type I.
